# Quantitative dissection of healthy aging using protein and peptide signatures from paired CSF and plasma

**DOI:** 10.1002/alz.089108

**Published:** 2025-01-09

**Authors:** Polina Shichkova, Aida Kamalian, Marco Tognetti, Christopher Below, Roland Bruderer, Yuehan Feng, Michael W Lutz, Abhay Moghekar

**Affiliations:** ^1^ Biognosys AG, Schlieren, Zurich Switzerland; ^2^ Johns Hopkins University School of Medicine, Baltimore, MD USA; ^3^ Duke University School of Medicine, Durham, NC USA

## Abstract

**Background:**

Despite age being the primary risk factor for Alzheimer's disease (AD), there remains a necessity for a thorough understanding of the distinct biological pathways affected in the course of healthy aging as opposed to the pathological aging that leads to neurodegeneration. As the genome remains constant throughout one's lifespan, it becomes crucial to unravel the impact of aging on the proteome. Proteins, being key players in various cellular functions, mediate the effects of environmental stimuli and epigenetic alterations. Moreover, despite the brain's protection by the blood‐brain barrier, emerging evidence suggests that systemic events like inflammation and metabolic dysfunction can significantly influence the risk of developing AD.

**Method:**

Matched CSF and plasma samples were obtained from individuals at the same visit from the Neurology Clinic and Alzheimer’s Disease Research Center at Johns Hopkins. Samples were collected from young control subjects (n= 53), and healthy elderly subjects (n = 40). The plasma samples were depleted in 96‐well format using an automated MARS‐14 depletion system. The depleted plasma and neat CSF samples were subsequently processed to tryptic peptides and analyzed using data‐independent acquisition (DIA)‐mass spectrometry (MS). Data processing and analysis were performed using Biognosys’ Spectronaut software.

**Result:**

Using our optimized discovery workflow, we analyzed the above‐described cohort of 93 matched plasma and CSF sample pairs. This resulted in more than 73254 quantified peptides (associated with > 4000 proteins) in CSF and 62750 quantified peptides (associated with > 2500 proteins) in plasma. The depth and breadth of protein quantification cover numerous pathological mechanisms such as AB and Tau pathology, synaptic dysfunction, neuronal injury, iron toxicity and inflammation. Harnessing the unique feature of MS‐based proteomics, we focused on the identification of differentially regulated peptides that are located in protein regions where known events such as alternative splicing, truncation or protease cleavage occur.

**Conclusion:**

The peptide‐centric nature of our approach allowed us to assess a variety of post‐translational modifications, truncations and isoforms. We envision this unbiased profiling of proteoforms as a powerful tool to elucidate complex disease mechanisms.

